# Melatonin and Vitamins: A Promising Combination to Augment Conventional Anticancer Therapies

**DOI:** 10.3390/nu17193120

**Published:** 2025-09-30

**Authors:** Wamidh H. Talib, Suha M. Sabri, Rawan W. Hadi, Viktória Prémusz, Tamás Beregi

**Affiliations:** 1 Faculty of Allied Medical Sciences, Applied Science Private University, Amman 11931-166, Jordan; 2 Department of Clinical Nutrition and Dietetics, Applied Science Private University, Amman 11931-166, Jordan; s_sabri@asu.edu.jo (S.M.S.); rawanalyasari2001@gmail.com (R.W.H.); 3 Institute of Physiotherapy and Sports Science, Faculty of Health Sciences, University of Pécs, H-7621 Pécs, Hungary; premusz.viktoria@pte.hu (V.P.); tamas.beregi@etk.pte.hu (T.B.); 4 Physical Activity Research Group, János Szentágothai Research Center, National Laboratory on Human Reproduction, University of Pécs, H-7624 Pécs, Hungary

**Keywords:** combination anticancer therapy, vitamin C, vitamin D, vitamin E

## Abstract

Cancer remains a major global health challenge, requiring new adjunctive therapies. Integrative oncology, which combines conventional treatments with complementary agents, has gained attention for improving patient outcomes. Melatonin, a potent antioxidant and immunomodulator, has shown promise in cancer therapy. Recent evidence suggests that combining melatonin with vitamins—particularly vitamin D, vitamin C, and vitamin E—may enhance its anticancer effects through synergistic mechanisms. Melatonin exerts anticancer effects by regulating oxidative stress, apoptosis, and immune responses. Vitamin D enhances immune modulation, while vitamins C and E provide antioxidant and cytoprotective benefits. Their combined action may improve tumor suppression and reduce treatment-induced toxicity. However, despite promising preclinical data, clinical studies on melatonin–vitamins synergy remain limited. This review explores the molecular interactions, current evidence, and research gaps in melatonin–vitamin combinations for cancer therapy. Future studies should focus on mechanistic insights, optimal dosing, and clinical trials to establish their role in integrative oncology. Unlocking this potential could enhance existing cancer treatment strategies and improve patient outcomes.

## 1. Introduction

Cancer is a chronic disease and considered a global health problem. The incidence of this disease is continuously rising, and global statistics estimate a number of 27.5 million new cancer cases every year by 2040 [1].

Cancer patients are mainly treated with conventional protocols, including chemotherapy, radiotherapy, and surgery. However, these treatments are often associated with certain drawbacks, including high toxicity, the development of resistance, and incomplete tumor eradication [2]. Due to these limitations, complementary and alternative therapies have attracted increasing interest in scientific research. Among these, the combination of melatonin and vitamins has recently emerged as a particularly promising strategy to enhance the efficacy of conventional anticancer therapies [3].

Melatonin is a hormone produced mainly by the pineal gland and functions in sleep–wake cycle control. Epidemiological studies revealed a direct link between night shift workers and increased risk of cancer. Experimentally, melatonin caused inhibition in cancer proliferation and reduced angiogenesis associated with disturbances in circadian rhythm. This inhibition was mediated by targeting multiple genes, including *p53*, *TNF-α*, *Per2*, *VEGF-A*, and *PDGF-C* [4]. Melatonin was also reported to inhibit metastasis through targeting MMP2 and MMP9, which are involved in tissue invasion [5]. Immune evasion is one of the mechanisms used by cancer cells to inhibit the anticancer immune response. Melatonin was observed to enhance the anticancer immune response through activation of T lymphocytes and suppression of PD-L1 expression [6].

Similarly, numerous epidemiological and preclinical studies support the role of vitamins such as vitamin A, C, D, and E in cancer prevention and treatment. Both vitamin A and C were reported to induce the expression of NK group 2D ligands (NKG2DLs) and facilitate the binding of NK cells with tumor cells. Such binding is essential for the elimination of cancer cells by NK cells [7]. Additionally, vitamin D inhibited gastric cancer cells through downregulation of CD44, which is a cancer stem cell marker [8].

Interestingly, combining vitamins with melatonin has recently emerged as an exceptionally promising strategy that may significantly potentiate the efficiency of conventional anticancer therapies. Some vitamins share similar mechanisms of action with melatonin, acting through multiple pathways due to their effects in reducing inflammation and oxidative stress.

This review discusses recent studies on the combined use of melatonin and vitamins in cancer therapy. It highlights their synergistic effects, mechanisms of action, and potential clinical value in enhancing treatment efficacy and improving patient outcomes.

## 2. Melatonin in Cancer Therapy

**Figure 1:**
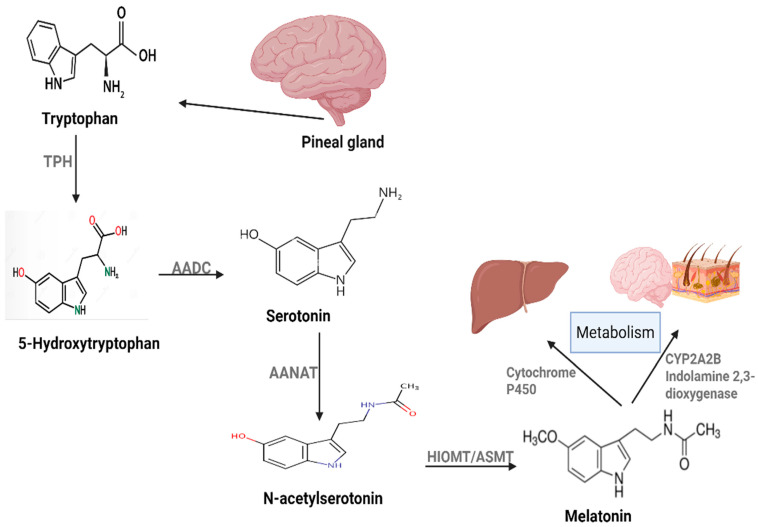
Melatonin production and metabolism in human body.

Melatonin (N-acetyl-5- methoxytryptamine) is an indole hormone regulating various physiological processes, including the circadian rhythm. It is mainly produced by the pineal gland in response to darkness. Its production is mediated by a synthetic mechanism involving tryptophan and serotonin (Figure 1), but it is also produced in the brain, skin, bone marrow, lymphocytes, retinas, and the gastrointestinal tract. Melatonin is mainly metabolized in the liver by cytochrome P450 enzymes. However, other tissues are also involved in melatonin metabolism. These tissues include the skin and brain using either CYPA2B or 2,3-indolamine dioxygenase [9]. The role of melatonin in cancer therapy involves different mechanisms, ranging from indirect effects such as antioxidant and immune modulation to more direct mechanisms, including apoptosis induction, angiogenesis inhibition, and anti-metastasis [10]. Below we will discuss the signaling pathways and molecular targets involved in melatonin’s anticancer activity.

### 2.1. Antioxidant Properties and Mitochondrial Function

Several studies have shown that melatonin has antioxidative effects, providing protection against damage from carcinogenic substances and acting as a free radicle scavenger. The antioxidant activity of melatonin is related to its ability to neutralize reactive nitrogen (RNS) and oxygen (ROS) species [11]. The antioxidant activity of melatonin also involves upregulation of antioxidant enzymes, including superoxide dismutase (SOD), catalase (CAT), glutathione peroxidase (GPx), and peroxidase (POX). The antioxidant properties of melatonin continue even after metabolism [12]. A range of experimental data showed that melatonin can generate several antioxidant metabolites [13].

Additionally, melatonin enhances mitochondrial function by acting as a potent antioxidant, reducing oxidative stress, and protecting mitochondria from damage. It promotes mitochondrial biogenesis, increases ATP production, stabilizes mitochondrial membranes, and improves energy efficiency by enhancing the function of the electron transport chain (ETC), reducing electron leakage, and minimizing free radical generation. Furthermore, melatonin regulates mitochondrial dynamics by modulating fission and fusion processes and preventing cell death through apoptosis. Synthesized locally within the mitochondria of non-pineal cells, melatonin is primarily used for metabolic regulation and can act through autocrine and paracrine mechanisms. Even in the absence of the pineal gland, mitochondrial melatonin levels remain unaffected, as key enzymes like AANAT and ASMT facilitate its synthesis [14,15].

Moreover, melatonin reprograms cancer cell metabolism by inhibiting pyruvate dehydrogenase kinase (PDK), thereby redirecting pyruvate into the mitochondria for oxidative phosphorylation. This metabolic shift reduces glycolysis, increases oxidative stress, and induces apoptosis, providing potential insights into combating Warburg effect-dependent cancer growth and improving energy production and cellular function [14,15].

### 2.2. Anticancer Mechanisms of Melatonin

Melatonin exhibits multiple anticancer properties. It has been reported to induce apoptosis, inhibit tumor cell proliferation, and suppress angiogenesis. In addition, melatonin exerts tumor-suppressive effects through its antioxidant activity and immune system through the stimulation of immune cells that are involved in anticancer activity and the inhibition of T-regulatory cells (Tregs) and cancer-associated fibroblasts (CAFs) [16].

**Figure 2:**
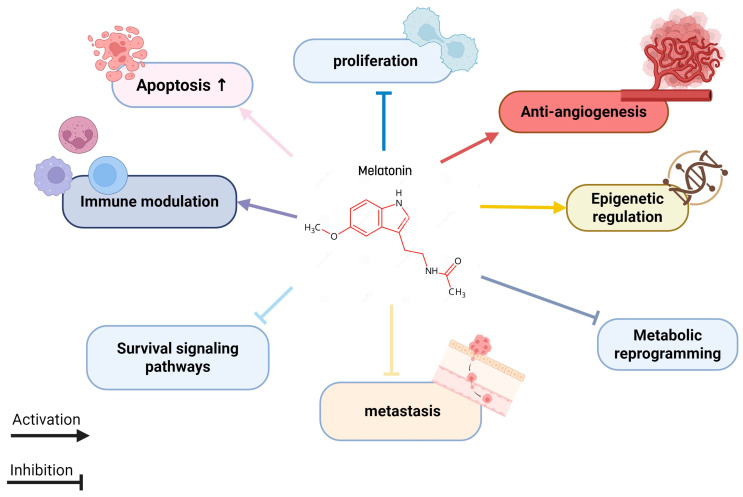
Melatonin targeting multiple cancer hallmarks.

Moreover, various studies have shown that melatonin can interfere with tumor progression by influencing survival signaling pathways, inhibiting metastasis, and regulating epigenetic mechanisms. It also attenuates the metabolic reprogramming of cancer cells (Figure 2). These anticancer effects are closely related to the chemical structure of melatonin, as it belongs to the acetamide group, specifically an acetamide where a hydrogen atom on the nitrogen is replaced by a 2-(5-methoxy-1H-indol-3-yl) ethyl group. Interestingly, other compounds from the acetamide family have also been reported to possess anticancer properties [17].

### 2.3. Immune Modulation and Inflammatory Regulation

In addition to direct anticancer effects, melatonin regulates immune function and cancer through complex mechanisms involving its receptors on immune cells. It enhances immune responses by increasing the activity of macrophages, microglia, natural killer (NK) cells, and monocytes. Melatonin also stimulates the production of interleukins such as IL-1, IL-6, and IL-12 by monocytes and promotes T helper cell activation, thereby accelerating the adaptive immune response. Additionally, it boosts leukocyte chemotaxis, antigen presentation, and cytokine production (e.g., IL-1β, IL-6, TNF-α) [18,19,20].

**Figure 3:**
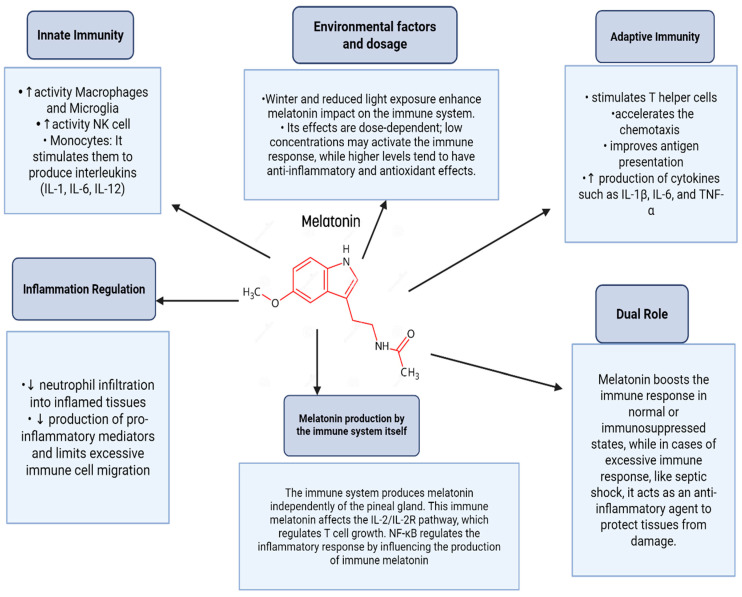
Role of melatonin in immune system modulation.

Moreover, melatonin modulates inflammation by reducing neutrophil infiltration, pro-inflammatory mediators, and immune cell migration during excessive immune activation. Notably, melatonin plays a dual role: it acts as an immunostimulant in basal or immunosuppressed states, enhancing defenses against infections and tumors, while serving as an anti-inflammatory agent during excessive immune activation, such as in septic shock [18,19,20] (Figure 3).

The immune system itself can produce melatonin, which interacts with IL-2/IL-2R signaling and is regulated differently from pineal-derived melatonin, with NF-κB playing distinct roles in this inflammatory regulation. Environmental factors, such as winter darkness, can influence the immune effects of melatonin, and its dose-dependent actions further highlight its regulatory complexity. With minimal side effects being observed in experimental models, melatonin shows promise as an adjunct therapy for viral infections and other conditions requiring immune modulation [18,19,20].

### 2.4. Epidemiological and Clinical Evidence

Substantial epidemiological data have demonstrated the role of melatonin in cancer prevention. In a case–control study involving female participants, the results showed that females with low serum melatonin (≤39.5 pg/mL) had a higher risk of breast cancer compared with other females with higher melatonin levels (˃39.5 pg/mL) [21]. Similarly, a retrospective study found that the serum melatonin levels in women with ovarian cancer were significantly lower compared with control subjects, indicating that reduction in circulating melatonin levels might contribute to the pathogenesis of ovarian cancer [22]. Borin et al. [23] confirmed a reduction in breast cancer proliferation after treatment with melatonin. Additionally, melatonin showed anti-metastatic activity by inhibiting Rho-associated kinase protein. Furthermore, enhanced anticancer activity was reported when melatonin was used in combination with chemotherapy. The combination of melatonin with taxol inhibited cancer metastasis by targeting the DJ-1/KLF17/ID-1 signaling pathway [24].

Previous studies showed high potential of melatonin to inhibit gastric cancer using multiple pathways. In one study, HIF-1a and VEGF production were inhibited after treatment with melatonin. This inhibition is mediated through targeting different signaling pathways, resulting in gastric cancer inhibition [25]. Angiogenesis inhibition was also reported as an anticancer mechanism of melatonin in gastric cancer. This inhibition was achieved through lowering the expression of VEGF mRNA in SGC-7901 gastric cancer cells [22].

### 2.5. Clinical Applications and Synergistic Effects

The use of melatonin in combination with other chemotherapeutics is the main strategy of using melatonin clinically. Enhanced therapeutic activity and reduced toxicity were observed after using melatonin in combination with chemotherapy. Also, improvements in sleep and life quality were observed in these combination therapies [26].

A synergistic effect was reported for combinations consisting of melatonin and antioxidants, including vitamin C, carotenoids (provitamin A), tocopherols (vitamin E), and vitamin D [27].

## 3. Vitamins as Anticancer Agents

Vitamins are important micronutrients for maintaining physiological processes and are considered crucial agents in cancer prevention and therapy [27]. In recent years, studies have shown that vitamins have potent anticancer properties, which depend on diverse mechanisms. These include their antioxidant capacity to neutralize free radicals and, importantly, their roles in DNA repair and maintaining genomic stability. Additionally, vitamins regulate immune responses and induce apoptosis in cancer cells. These mechanisms strongly support the use of vitamins in suppressing the carcinogenesis and highlight their potential in developing therapeutic strategies for cancer prevention and treatment [28,29,30].

### 3.1. Mechanisms of Action of Vitamins in Cancer

Vitamins exhibit multiple mechanisms regulating their anticancer effects, showing their functions in cancer biology. In addition, vitamins can play a vital role as antioxidants by neutralizing reactive oxygen species (ROS) and reducing oxidative stress, which is an important factor contributing to cancer progression and DNA damage [31]. Moreover, vitamins effectively contribute to DNA repair by reducing the development of tumors caused by mutagenic episodes [27]. They can modulate immune cell activity by enhancing immune system activity to recognize and eliminate cancer cells. Importantly, there are specific types of vitamins that help in regulating gene expressions and cellular signaling pathways, which induces apoptosis and inhibits the proliferation of the uncontrolled cancer cells [32,33].

#### 3.1.1. Vitamin D

Vitamin D is a hydrophobic molecule that is dissolved in fat and involved in multiple functions, including calcium absorption and homeostasis. Immunomodulation and antioxidant and anti-inflammatory effects were also suggested as biological activities of vitamin D [34].

The link between vitamin D and cancer was reported in several studies. An increase in the risk of cancer development is associated with low vitamin D levels in the blood [35]. An interesting study showed that exposure to sunlight during summer was associated with less aggressive cancer. The results of this study were supported by experimental and epidemiological findings that showed decreased proliferation in cancer cells when exposed to high concentrations of vitamin D [36]. Likewise, a significant association was reported between factors that reduce vitamin D and cancer incidence. Such associations support the protective effect of vitamin D against various types of cancers [35].

Vitamin D’s anticancer effect involves an indirect mechanism through immune system modulation. In this context, vitamin D was reported to activate various immune cells such as CD4 T cells, macrophages, monocytes, and antigen-presenting cells [37]. Proliferation and differentiation of T lymphocytes are modulated by the active form of vitamin D (1,25(OH)_2_D). The activation of dendritic cells and shifting cytokine expressions are additional mechanisms for vitamin D’s effect on the immune system [38].

Consistent with these findings, McDonnell et al. [39] found an association between decreased breast cancer risk and high concentrations of 25(OH)D, with the most protective concentration being 60 ng/mL and above. Such a protective effect is due to multiple mechanisms. Binding active vitamin D to its receptor on normal breast tissue induces cell differentiation, increases cell adhesion, reduces DNA damage, and suppresses inflammation. All these effects may contribute to cell protection and reduce the rate of transformation into cancer cells [40]. On the other hand, the binding of vitamin D on breast cancer cells triggers apoptosis and stimulates other mechanisms that slow down cell division [40]. 

**Figure 4:**
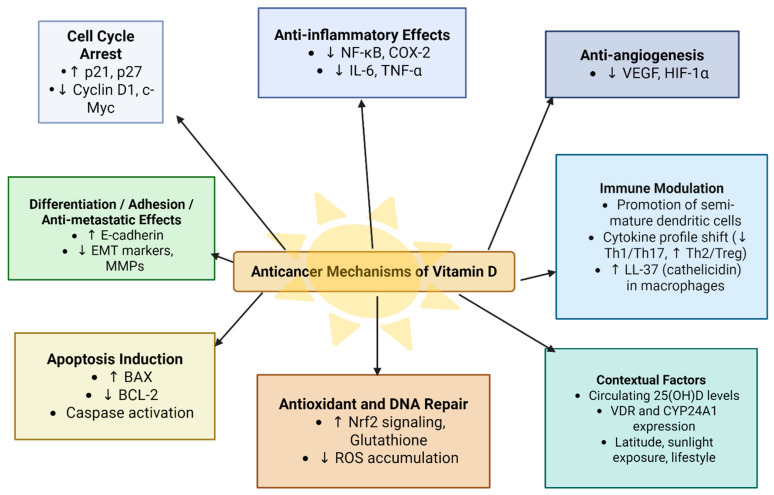
Anticancer mechanisms of vitamin D.

Other metabolites of vitamin D such as 20(OH)D3 also showed protective effects against cancer development [41]. Figure 4 summarizes the anticancer mechanisms of vitamin D.

#### 3.1.2. Vitamin C

Vitamin C is a hydrophilic molecule with a hydroxylated lactone ring. This chemical structure makes vitamin C an efficient donor for protons and electrons, which is important for its ability to decrease reactive oxygen species levels. Because of its antioxidant effects, vitamin C plays an important role in protecting cells from oxidative stress, and it is therefore considered an anticancer agent [33].

An inverse correlation between cancer incidence and vitamin C intake was observed in epidemiological studies. In one study on breast cancer, a reduced risk of cancer development was associated with higher plasma levels of vitamin C [42]. Another study reported a significant association between vitamin C intake and reduced mortality among breast cancer patients [43]. Moreover, vitamin C was also tested as a therapy through intravenous injection at pharmacological doses. The results of this study showed inhibition of cancer cells, without toxicity on normal cells [44]. Additionally, the use of vit C in combination with chemotherapy caused a reduction in the side effects associated with chemotherapy [45,46].

**Figure 5:**
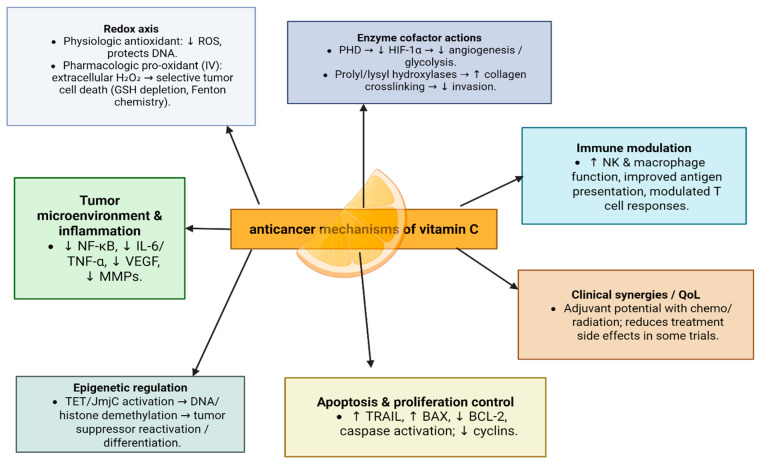
Anticancer mechanisms of vitamin C.

The anticancer effect of vit C is mediated by multiple molecular mechanisms, including decreasing the entry of ROS inside cells, reducing metastasis through stabilizing collagen crosslinking in the extracellular matrix, and altering the gene expression by breaking down hypoxia-inducible factors [47] (Figure 5).

In vitro studies have also demonstrated that vitamin C can promote apoptosis in breast cancer cells by increasing TRAIL expression [48].

In addition, vitamin C may prevent cancer by modulating various biological processes. Vitamin C is a critical cofactor for many groups of hydroxylases that are involved in controlling the transcription factor hypoxia-inducible factor 1 (HIF-1). Elevated HIF levels promote tumor growth and development, but with the opposing hydroxylases present, HIF can be managed to prevent tumorigenesis [49].

Additionally, vitamin C has been shown to regulate metabolic pathways and cellular signaling, enhancing the effectiveness of conventional cancer therapies when used as an adjuvant treatment. Ongoing research aims to optimize the clinical application of vitamin C in combination with conventional therapies [50].

#### 3.1.3. Vitamin E

Vitamin E is a lipid-soluble antioxidant composed primarily of tocopherols and tocotrienols, which have a critical role in protecting cellular membranes from oxidative damage [51]. These compounds act as effective free radical scavengers, inhibiting lipid peroxidation and reducing ROS-induced DNA damage [52]. Among the forms of vitamin E, tocotrienols have shown stronger anticancer activity in comparison with tocopherols due to their greater ability to suppress angiogenesis and tumor growth [51].

**Figure 6:**
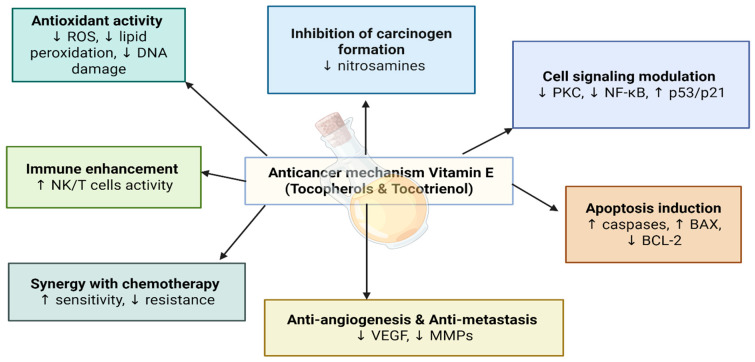
Anticancer mechanisms of vitamin E.

In addition to its antioxidant role, vitamin E has the ability to influence signaling pathways with cell growth and cellular apoptosis, further enhancing its anticancer efficacy [52]. For instance, alpha-tocopherol has shown the ability to inhibit endogenous nitrosamine or nitrosoamide formation, suppress cell proliferation, inhibit tumor angiogenesis, and enhance immunity [53] (Figure 6).

Several epidemiologic studies have shown that low levels of vitamin E are associated with an increased risk of specific cancers, including breast, lung, and esophageal cancers [53,54,55]. For example, it was found that higher vitamin E levels were related to decreased lung cancer risk over a 28-year period [53]. Additionally, a meta-analysis showed that higher vitamin E intake is negatively associated with the risk of esophageal cancer [55].

Furthermore, combining tocopherols or tocotrienols with chemotherapeutic agents such as methotrexate or tamoxifen has been shown to enhance anticancer activity and the inhibition of cancer cell growth [56,57].

#### 3.1.4. Vitamin A and Retinoids

Vitamin A is known as an effective regulator of cell differentiation and proliferation. It has many derivatives, particularly retinoids, which have critical roles and properties in cellular function [58]. The mechanism of action of retinoids is explained by binding to retinoic acid receptors and retinoid X receptors. These interactions influence gene expressions that are involved in the cell cycle and regulation of apoptosis [59]. Many studies have shown that having these properties can make retinoid more effective in therapeutic strategies against many cancers such as acute promyelocytic leukemia (APL) and skin cancers [59,60].

In addition to their therapeutic effects, retinoids also have chemopreventive properties, which help in maintaining and regulating the epithelial integrity and preventing the transformation of dysplastic cells [59,60]. Through these mechanisms, retinoids have the ability to prevent the recurrence of the cancer and can selectively induce apoptosis in cancer cells with their therapeutic potential [58].

#### 3.1.5. B Vitamins

B vitamins, particularly folate (Vitamin B9) are essential cofactors that have many types in one carbon metabolism, which is an important process, especially for synthesizing DNA and repairing it [61]. Low levels of folate have been associated with genomic instability and increased DNA mutations, which elevate the risk of cancers, particularly those involving rapidly dividing tissue such as colon and cervix cancer [62]. Folate plays an important role in nucleotide synthesis and methylation processes that are capable of enhancing the function of tumor suppressor genes and reduce the risk of mutations associated with oncogenic processes [63]. Recent studies suggest that folate supplementation can help in reducing the growth of specific cancer types. However, strict supervision and evaluation are necessary in order to reduce the risks associated with excessive intake, which could promote the growth of certain types [63].

The anticancer potential of vitamins is an area of active investigation, offering promising avenues for cancer prevention and treatment [64]. By elucidating the intricate mechanisms by which vitamins modulate oxidative stress, genomic stability, immune responses, and cellular signaling, researchers are unlocking their therapeutic potential. The integration of vitamin-based strategies into conventional cancer therapies could revolutionize cancer care, paving the way for more effective and personalized treatment regimens [65].

## 4. Combination of Melatonin and Vitamins: A Possible Synergistic Effect

**Figure 7:**
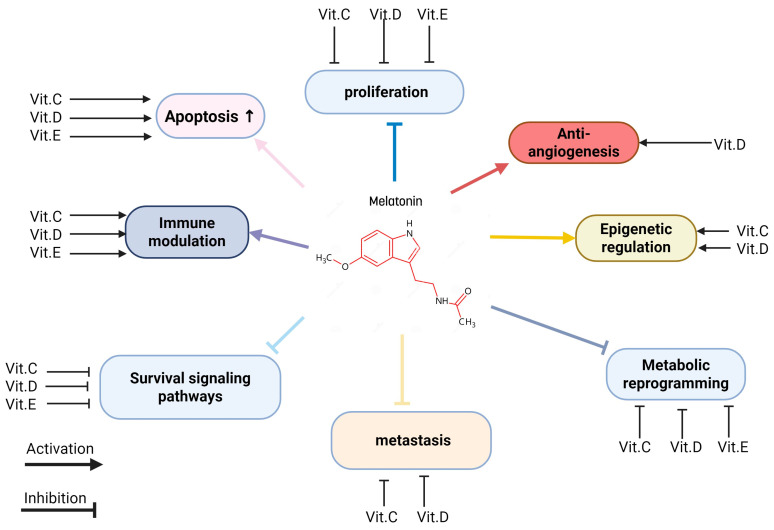
Integrated anticancer mechanisms of melatonin and vitamins.

The combination of melatonin and various vitamins can have a significant synergistic effect on health; some vitamins share a mechanism of action that is similar to that of melatonin as an anticancer, like anti-inflammatory and antioxidant activities [8]. Some highlighted combinations are melatonin with vitamin D, vitamin C, folic acid, and vitamin E (Figure 7).

### 4.1. Melatonin and Vitamin D

Evidence from previous studies showed that both melatonin and vitamin D have multiple signaling pathways for targeting cancer. For example, melatonin was reported to inhibit cancer and reduce the adverse effects of conventional therapies [66]. These effects are also shared with vitamin D, and both agents were shown to be active in antiangiogenesis, antiproliferative, and anti-inflammatory mechanisms [65,66,67].

Melatonin and vitamin D can engage in cooperative action in the mitochondria, as both of them have targets in this organelle [68]. Both are involved in shared signaling mechanisms in mitochondria. The downregulation of mTOR, FOXO1, iNOS, NF-κB, and RAAS, as well as the upregulation of Klotho, SIRT-, AMPK, Nrf2, and HSP70, were reported as shared mechanisms for both agents. Furthermore, melatonin can bind to the vitamin D receptor (VDR), enhancing vitamin D signaling and cellular activity, which may enhance vitamin D’s anticancer activity [69]. The combination has also been shown to increase the release of TGF--β1, which activates the apoptotic cascade and reduces breast cancer cell enlargement. These findings indicate a strong synergistic potential for the combination of melatonin and vitamin D in anticancer studies [69], which indicates that melatonin and vitamin D carry out a lot of their functions together.

The beneficial effect of a synergistic therapy using melatonin and vitamin D in cancer patients was reported in many studies [70,71]. Bizzarri et al. [72] treated estrogen-responsive rat breast cancer cells using a combination of vit D and melatonin. The study showed that melatonin increased the sensitivity of RM4 cells to vitamin D3, and the combination treatment enhanced the release of TGF-β, which enhanced the growth inhibition of breast cancer cells and induced apoptosis. Moreover, the combination of melatonin and vitamin D3 significantly reduced Akt phosphorylation and MDM2 levels, with a consequent increase in the p53/MDM2 ratio [71]. Similar findings were also demonstrated by another study, which showed that the combined treatment of melatonin and vitamin D3 caused inhibition in MCF-7 breast cancer cells and induced apoptosis by upregulating the p53 gene expression. The study concluded that treatments with melatonin and vitamin D3 were found to have anticancer effects and could potentially prove to be the ideal natural adjuvant therapy for the treatment of breast cancer [73].

In another study, Ozerkan et al. [74] evaluated the hepato-protective activity of melatonin and vitamin D on CCl4-induced cytotoxicity in human hepatoma. The study showed that co-administration of melatonin and vitamin D3 protected liver cells from oxidative damage by diminishing lipid peroxidation and enhancing glutathione levels in a similar way to that of the control groups.

Several clinical studies have investigated the use of melatonin and vitamins, including vitamin D, as therapeutic agents in patients with breast, head, and neck cancer. One study showed that melatonin, when combined with somatostatin, retinoids, vitamin D3, and a low dose of cyclophosphamide, had positive effects in terms of efficacy and survival of patients with breast cancer in humans [75]. More recently, a retrospective observational study evaluated the effectiveness of treating patients with malignant anaplastic brain cancer using a combination of melatonin and several vitamins, including vitamin D. The study showed antitumor effects of the treatment and an increased survival rate among patients [76]. Aligned with these findings, Bella et al. [77] observed an improved survival rate in patients with osteosarcomas who were treated with a multi-therapy composed of melatonin and vitamin D, without overt toxicity.

### 4.2. Melatonin and Vitamin C

Melatonin and vitamin C are both potent antioxidants, and their combination has shown synergistic effects in anticancer activity. One of the most notable derivatives of vitamin C is accorbyl palmitate (AP), which acts as an antioxidant molecule and exerts antitumor activity via its antiproliferative effect. A study by El-Far et al. [78] showed that the combination of melatonin and ascorbyla palmitate-loaded pluronic nanoparticles (APnps) can synergistically reduce tumor growth, accompanied by increased antioxidant profiles, decreased levels of oxidative stress, and increased apoptosis and DNA damage. In addition, the combination of melatonin and APnps was able to inhibit cancer cell invasion and metastasis by decreasing the protein expression of MMP 9.

In another study, the combination of melatonin and vitamin C was found to reduce the levels of malondialdehyde (MDA) and myeloperoxidase (MPO), while increasing antioxidant enzymes such as superoxide dismutase (SOD), glutathione (GSH), and catalase (CAT) in the kidneys and heart, thereby reducing oxidative stress [79]. Furthermore, the combination lowered inflammatory markers like pro-calcitonin, presepsin, and pro-inflammatory cytokines, indicating a strong anti-inflammatory effect. These findings suggest that the combination of the two molecules could increase antioxidative and anti-inflammatory activities [80].

### 4.3. Melatonin and Vitamin E

Vitamin E is also a strong antioxidant, which indicates an important potential in cancer therapy, particularly when combined with melatonin. One study studied the effect of this combination on homocysteine (Hcy)-induced apoptosis in human umbilical vein endothelial cells (HUVECs) by testing antioxidant markers like reactive oxygen species (ROS) and lipid peroxidation (LPO) levels. The results showed that the combination reduced both ROS and LPO levels, which indicates a strong potential for anticancer activity [81].

## 5. Research Gaps and Future Directions

Although current studies demonstrate that melatonin and various vitamins share similarities, with a significant impact on human health, particularly through their anti-inflammatory and antioxidant actions, further research is needed before these results can be applied in clinical practice. One of the main gaps is that clinical trials on this combination are still limited to confirming the safety and efficacy of these combinations in cancer patients.

Another important consideration is the optimal dosage, as high doses may cause side effects or interact negatively with other treatments. Therefore, further studies are required to determine optimal doses when these agents are used in combination.

Furthermore, more studies are needed to better understand the mechanisms through which melatonin and these vitamins influence cancer growth, immune responses, and other pathways in the body.

Finally, to determine the efficacy of both agents in improving the life quality of patients with cancer, large controlled trials comparing the effects of combined therapy with melatonin and various vitamins with single agents are warranted, in order to investigate their potential synergistic effects.

## 6. Conclusions

The combined use of melatonin and vitamins is an appealing option for improving anticancer therapies. According to several sets of research evidence, melatonin has a pleiotropic mechanism for anticancer action through its antioxidant, immune-modulatory, and pro-apoptotic effects; this therefore suggests an effective adjuvant therapy. Several other vitamins have also been reported for cancer prevention and treatment due to their antioxidative and immunomodulatory roles: vitamin A, vitamin C, vitamin D, and vitamin E.

It is, therefore, proposed that the synergistic potential of melatonin combined with vitamins represents a holistic approach to cancer management, acting on several aspects of tumor biology while reducing the toxicities associated with conventional treatments. However, further research and clinical trials are needed to establish standardized protocols or their validation in various types of cancers. The integration of these natural compounds into the treatment of cancer can help in opening a way toward more efficient, less toxic, and more patient-oriented therapeutic approaches.

## Data Availability

The data presented in this study are available on request from the corresponding author.

## Abbreviations

**Abbreviation**	**Full Name**
25(OH)D	25-hydroxyvitamin D
AADC	Aromatic L-amino Acid Decarboxylase
AANAT	Arylalkylamine-N-acetyltransferase
Akt	Protein Kinase B
AP	Ascorbyl Palmitate
APL	Acute Promyelocytic Leukemia
APnp	Ascorbyl Palmitate-loaded Pluronic Nanoparticles
BAX	Bcl-2-associated X protein
BCL-2	B-cell lymphoma 2 protein
c-Myc	Myc proto-oncogene protein
CAFs	Cancer-Associated Fibroblasts
CAT	Catalase
CCl4	Carbon Tetrachloride
CD 44	Cluster of Differentiation 44
CD 4 T cells	Cluster of Differentiation 4 T cells
COX-2	Cyclooxygenase-2
Cyclin D1	Cyclin D1 protein
CYP24A1	Cytochrome P450 Family 24 Subfamily A Member 1
CYP2A2B	Cytochrome P450 Family 2 Subfamily A2B
DJ-1	Protein Deglycase DJ-1
E-cadherin	Epithelial Cadherin (Cell Adhesion Protein)
EMT	Epithelial–Mesenchymal Transition
ETC	Electron Transport Chain
FOXO1	Forkhead Box Protein O1
GPx	Glutathione Peroxidase
GSH	Glutathione
Hcy	Homocysteine
HIF-1α	Hypoxia-Inducible Factor 1 alpha
HIOMT/ASMT	Hydroxyindole-O-methyltransferase/Acetylserotonin O-methyltransferase
HUVECs	Human Umbilical Vein Endothelial Cell
ID-1	Inhibitor of DNA Binding 1
IL-1	Interleukin-1
IL-12	Interleukin-12
IL-1β	Interleukin-1 beta
IL-2	Interleukin-2
IL-2R	Interleukin-2 Receptor
IL-6	Interleukin-6
iNOS	Inducible Nitric Oxide Synthase
JMjc	Jumonji C
KLF17	Krüppel-like factor 17
LL-37	Cathelicidin Antimicrobial Peptide
LPO	Lipid Peroxidation
MDA	Malondialdehyde
MDM2	Mouse Double Minute 2 Homolog
MMP9	Matrix Metallopeptidase 9
MMPs	Matrix Metalloproteinases
MPO	Myeloperoxidase
mRNA	Messenger RNA
mTOR	Mammalian Target of Rapamycin
NF-κB	Nuclear Factor kappa-light-chain-enhancer of activated B cells
Nrf2	Nuclear factor erythroid 2–related factor 2
p21, p27	Cyclin-Dependent Kinase Inhibitors
p53	Tumor Protein p53
PDK	Pyruvate Dehydrogenase Kinase
PHD	Proly Hydroxylase Domain
PKC	Protein Kinase c
POX	Peroxidase
Qof	Quality of Life
RAAS	Renin–Angiotensin–Aldosterone System
RNS	Reactive Nitrogen Species
ROS	Reactive Oxygen Species
SGC-7901	Human Gastric Cancer Cell Line
SOD	Superoxide Dismutase
TET	Ten-Eleven Translocation
TGF-β1	Transforming Growth Factor Beta 1
Th1	T Helper Type 1 cells
Th17	T Helper Type 17 cells
Th2	T Helper Type 2 cells
TNF-α	Tumor Necrosis Factor-alpha
TPH	Tryptophan Hydroxylase
TRAIL	TNF-Related Apoptosis-Inducing Ligand
Tregs	T-Regulatory Cells
VDR	Vitamin D Receptor
VEGF	Vascular Endothelial Growth Factor
